# *Trans*-splicing of mRNAs links gene transcription to translational control regulated by mTOR

**DOI:** 10.1186/s12864-019-6277-x

**Published:** 2019-11-29

**Authors:** Gemma B. Danks, Heloisa Galbiati, Martina Raasholm, Yamila N. Torres Cleuren, Eivind Valen, Pavla Navratilova, Eric M. Thompson

**Affiliations:** 1 Sars International Centre for Marine Molecular Biology, University of Bergen, Bergen, Norway; 2 University of Ulm, Ulm, Germany; 3 Computational Biology Unit, Department of Informatics, University of Bergen, Bergen, Norway; 4 Inst Expt Bot, Czech Acad Sci, Centre of the Region Hana for Biotechnological and Agricultural Research, Olomouc, Czech Republic; 5 Department of Biological Sciences, University of Bergen, Bergen, Norway

## Abstract

**Background:**

In phylogenetically diverse organisms, the 5′ ends of a subset of mRNAs are *trans*-spliced with a spliced leader (SL) RNA. The functions of SL *trans*-splicing, however, remain largely enigmatic.

**Results:**

We quantified translation genome-wide in the marine chordate, *Oikopleura dioica,* under inhibition of mTOR, a central growth regulator. Translation of *trans*-spliced TOP mRNAs was suppressed, consistent with a role of the SL sequence in nutrient-dependent translational control of growth-related mRNAs. Under crowded, nutrient-limiting conditions, *O. dioica* continued to filter-feed, but arrested growth until favorable conditions returned. Upon release from unfavorable conditions, initial recovery was independent of nutrient-responsive, *trans*-spliced genes, suggesting animal density sensing as a first trigger for resumption of development.

**Conclusion:**

Our results are consistent with a proposed role of *trans*-splicing in the coordinated translational down-regulation of nutrient-responsive genes under growth-limiting conditions.

## Background

*Cis*-splicing of RNA in eukaryotes removes non-coding intronic sequences from protein coding mRNAs. This is essential to their translation. In a phylogenetically disparate group of organisms [1, 2] mRNAs also undergo *trans*-splicing [3] where a separately transcribed RNA molecule, called a spliced leader (SL), is added to their 5′ ends. An important function of this process is to resolve polycistronic RNA transcribed from operons, allowing their translation as monocistrons. Many non-operon, monocistronic transcripts, however, are also *trans*-spliced [4]. The function in these cases has so far remained largely enigmatic.

We previously proposed that the SL supplies a 5′ TOP-like nutrient-dependent translational control motif to *trans*-spliced mRNA [5, 6]. TOP mRNAs, which primarily encode the protein synthesis machinery, contain a conserved 5′ TOP (Terminal OligoPyrimidine) motif that is critical [7] for translational repression during unfavourable growth conditions. This translational repression reduces the large energy expenditure associated with protein synthesis.

Target of rapamycin (mTOR) [8, 9], a master regulator of growth, is conserved from yeast to human and selectively regulates the translation of mRNAs with a TOP or TOP-like motif [8]. As part of mTORC1 (one of two complexes containing mTOR), mTOR phosphorylates and represses the translational repressor, eukaryotic translation initiation factor 4E binding protein 1 (4E-BP1). Active 4E-BP1 binds eIF4E preventing the association of eIF4E with eIF4G, necessary for cap-dependent translation initiation, particularly of TOP mRNAs, which are more dependent on this association than other mRNAs [9]. Phosphorylated 4E-BP1 is unable to bind eIF4E and translation can proceed; mTOR thereby promotes translation of TOP mRNAs and its inhibition suppresses their translation.

In the urochordate, *O. dioica,* and the nematode, *C. elegans* the TOP motif is not encoded in the genome at the appropriate loci; classical TOP mRNAs in these species, therefore, lack a TOP motif. These TOP mRNAs are, however, *trans*-spliced with a pyrimidine-enriched SL sequence. The SL then forms the 5′ end where mTOR-dependent translational control is normally targeted at a TOP motif*.* These features suggest that SL RNAs contain TOP-like mTOR-dependent translational control elements [5]. This is further supported by our findings [5], and the findings of others [10], that *trans*-spliced mRNAs are enriched for growth-related functions. In addition, we previously found that the vast majority of maternal mRNAs in the three metazoan species we examined were *trans*-spliced [5]. In *O. dioica,* egg number is strongly dependent on nutrient levels [11] and is determined by partitioning a common cytoplasm into equally sized oocytes [12]. This gives further evidence of roles for both mTOR and *trans*-splicing in the control of maternal protein levels according to growth conditions.

Here, we tested this idea by using ribosome profiling to quantify translation genome-wide in female *O. dioica* treated with the mTOR inhibitor Torin 1 [9, 13]. The mTOR-regulated translatome was conserved between *O. dioica* and other species. Moreover, classical TOP mRNAs that possess a 5′ *trans*-spliced SL sequence rather than an encoded TOP motif were nevertheless subject to translational control via the mTOR pathway in *O. dioica*. These results suggest that *trans*-splicing may play a key role in the coordinated nutrient-dependent translational regulation of growth-related genes.

Under conditions of nutrient depletion and high animal density, *O. dioica* enters a developmental growth-arrested state (stasis), prior to the onset of meiosis, and mTOR activity is down-regulated [14] indicating that the translation of TOP mRNAs is suppressed. Once conditions become more favourable the animals recover and resume normal development [5, 14].

In the absence of food, the nematode *C. elegans* also enters a state of developmental growth arrest (L1 diapause). When food becomes available, animals resume development. In *C. elegans*, transcription of operons is preferentially up-regulated during recovery from growth arrest [10]. In *O. dioica*, however, it is instead non-*trans*-spliced monocistrons that are transcriptionally up-regulated during recovery from growth arrest [5]. We previously proposed that during recovery from growth arrest, *O. dioica* up-regulates TOP mRNAs and other *trans*-spliced transcripts via translational control, rather than transcriptional control, as a faster initial response [5]. Here, we tested this concept using ribosome profiling on *O. dioica* during growth arrest and recovery and found that, as with transcription, the translation of *trans*-spliced genes, including mTOR-targeted *trans*-spliced TOP mRNAs, were not preferentially up-regulated during recovery. This suggests that the primary, first response during recovery in *O. dioica,* is not mediated by coordinated, enhanced translation of *trans*-spliced SL mRNAs containing a TOP-like motif.

Together, our data support the proposal that *trans*-splicing in metazoans plays a key role in nutrient-dependent translational control.

## Results

### Profiling the mTOR-regulated translatome in *O. dioica*

In order to determine whether or not the translation of *trans*-spliced TOP mRNAs is regulated by mTOR in *O. diocia*, we profiled translation genome-wide using ribosome profiling with deep sequencing [15] in day 6 female animals that were exposed to either the mTOR inhibitor Torin 1 or DMSO control. At this developmental stage the bulk of the animal’s mass is from a single-celled coenocyst [12, 16, 17] within the ovary, the transcriptional output of which is enriched for *trans*-spliced transcripts [5]. In parallel, we sequenced total RNA in order to normalise ribosome protected RNA fragments (RPFs) to the abundance of mRNA transcripts (RNA), giving a measure of translational efficiency for each gene in both treatment and control conditions.

**Fig. 1:**
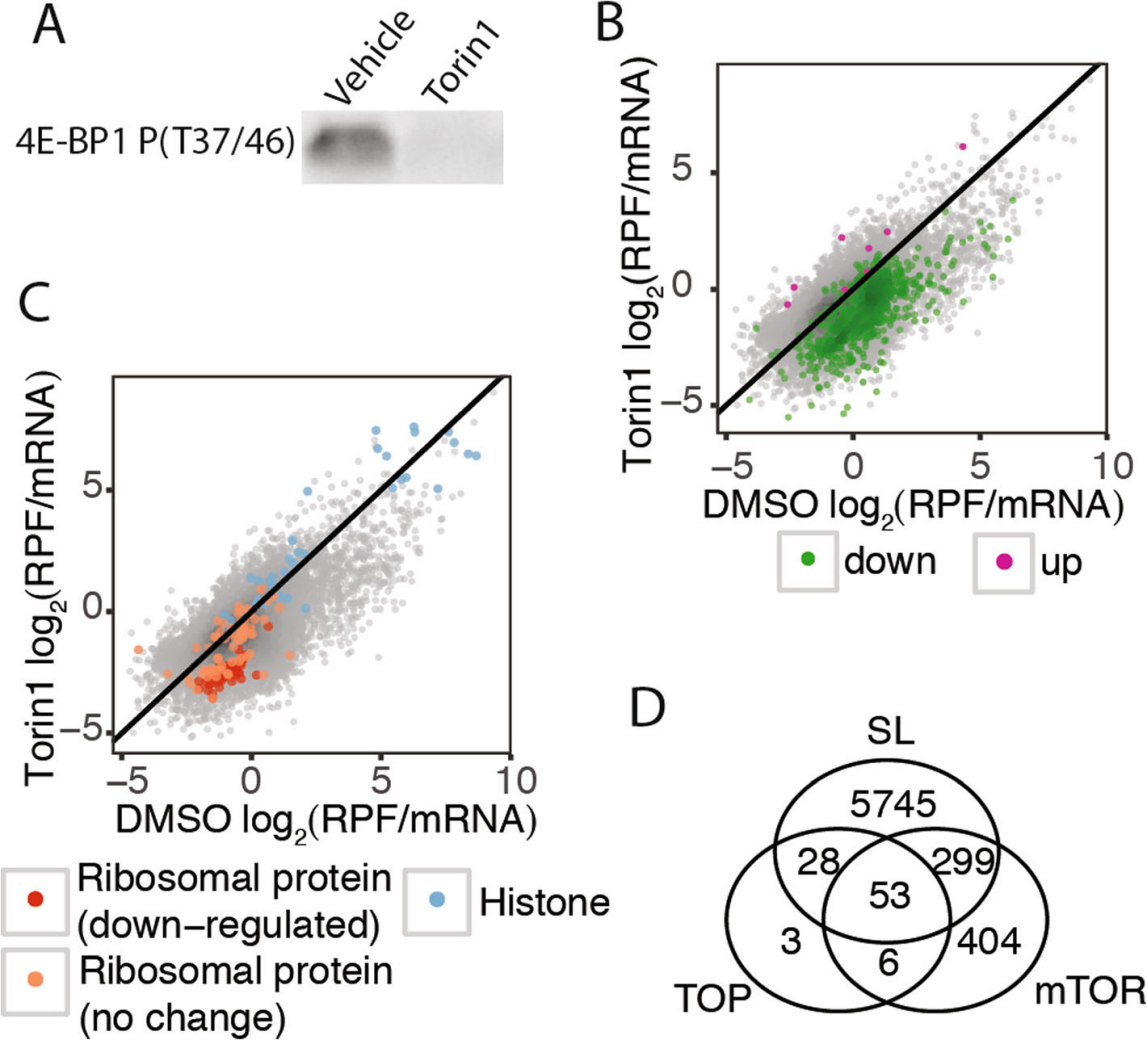
The mTOR-regulated translatome of *O. dioica*. **a** Adult animals were exposed to the mTOR inhibitor, Torin 1 (1 μM), or DMSO (vehicle control) in seawater for 1.5 h and female animals were collected. Phosphorylated 4E-BP1 was detected in DMSO but not in Torin 1 treated animals, confirming that mTOR was inhibited in Torin 1-treated animals (one out of three replicates shown; total protein used as a loading control and normalisation of band intensity; see Additional file 1: Figure S1D for full blot). **b** Median translational efficiency (RPF/mRNA = ribosome protected fragment density/mRNA density) of mRNAs from 3 replicates for Torin 1- and DMSO-treated animals with transcripts identified as having significantly up- or down-regulated translation highlighted. **c** Translational efficiencies shown as in (**b**) with known Torin 1-resistant (histone mRNAs) and Torin 1-sensitive (ribosomal protein mRNAs) gene categories highlighted to show that targets of mTOR-mediated translational control are conserved in *O. dioica*. **d** Intersections of orthologs of known TOP mRNAs (TOP), mTOR-regulated mRNAs (mTOR) and mRNAs that are *trans*-spliced (SL)

We confirmed that exposing female *O. dioica* to the mTOR inhibitor Torin 1, in seawater, resulted in the expected absence of phosphorylated 4E-BP1 (Fig. 1a and Additional file 1: Figure S1): phosphorylated 4E-BP1 was absent after 1.5 h of treatment, similar to what was observed in mouse embryonic fibroblast (MEF) cells [9]. We also demonstrated that the commercial antibody we used was specific to the phosphorylated form of 4E-BP1 in *O. dioica*, as it is in other species (Additional file 1: Figure S1E). In addition, we used polysome profiling to show a global down-regulation of translation as indicated by reduced polysome peaks (Additional file 2: Figure S2).

We measured the effect of mTOR inhibition on the translation of individual mRNAs by quantifying and precisely mapping RPFs and normalising to total RNA to obtain the translational efficiency (TE) of each mRNA. This allows the detection of mRNAs with unusually high or low ribosome density given their transcript abundance. Sequencing generated 57.9 M (vehicle control: DMSO) and 42.8 M (Torin 1) total RNA exon-mapped reads and 24.1 M (DMSO) and 1.4 M (Torin 1) RPF exon-mapped reads, across three biological replicates (Additional file 8: Table S1). By excluding genes with low read counts (see Methods) we were able to confidently assess the translational efficiency of 14,574 expressed genes out of 17,212 in the *O .dioica* reference genome. Our results indicate that the mTOR-regulated translatome is conserved between *O. dioica* and vertebrates. As expected, we found the set of genes regulated by mTOR was enriched for classical TOP mRNAs, the majority of which are *trans*-spliced in *O. dioica*.

### The mTOR-regulated translatome is conserved between *O. dioica* and mammals and enriched for TOP mRNAs that are SL *trans*-spliced in *O. dioica*

We detected 762 genes with mRNAs that had significantly reduced translational efficiencies when mTOR was inhibited (Fig. 1b and Additional file 9: Table S2). These represent the main targets of mTOR-mediated translational control in female *O. dioica*. Gene ontology (GO) analysis revealed that these were enriched for functions known to be regulated by TOR signalling, including translation and translation elongation, mitotic spindle elongation, fatty acid metabolism and TOR signalling (Additional file 3: Figure S3). Importantly, these include known TOP mRNAs: 60/127 expressed *O. dioica* ribosomal protein mRNAs (129 ribosomal proteins are annotated in the genome) and 59/89 mRNAs with orthologs to known human TOP mRNAs [18] (mostly ribosomal protein mRNAs) were significantly down-regulated (Fig. 1c, d and Additional file 10: Table S3, Additional file 9: Table S2). As found in mammalian cells [9], histone mRNAs were amongst those resistant to Torin 1 (Fig. 1c and Additional file 9: Table S2). We validated these results by qRT-PCR, testing 10 genes with the largest translational changes, which showed no statistically-significant changes in gene expression upon Torin 1 treatment (Welch Two Sample t-test, *p* > 0.05). The similarity of the mTOR-dependent translatome between *O. dioica* and mammals [9] indicates that the targets of mTOR regulation are likely conserved between *O. dioica* and it’s sister group, vertebrates. The down-regulation of translation of *trans*-spliced TOP mRNAs confirm that they are regulated by mTOR, indicating that the SL likely replaces the role of the critical TOP motif found in TOP mRNAs of other species.

### Translation of *trans*-spliced TOP mRNAs is regulated by mTOR

The TOP motif in vertebrate canonical TOP mRNAs (ribosomal proteins and other members of the translational apparatus) is highly conserved and required for growth-dependent translational control via mTOR signalling [7]. A 5′ TOP motif is also enriched in ribosomal protein mRNAs in the ascidian *Ciona intestinalis* [5, 19]. The canonical TOP motif begins with a cytosine and is followed by a stretch of 4–14 pyrimidines [20]. It was recently shown in MEFs that mTOR regulates a broader spectrum of mRNAs [9]. These are enriched for the presence of a TOP-like pyrimidine-enriched motif (a stretch of at least 5 pyrimidines within 4 nucleotides of the transcription start site (TSS)) [9]. The majority of established TOP mRNAs, including those discovered recently, are *trans*-spliced in *O. dioica* [5]. These include 103 out of all 129 (80%) annotated ribosomal proteins (127 ribosomal proteins were expressed in day 6 females), 33 out of 40 eukaryotic translation initiation factors (including 4 out of 5 that are known TOP mRNAs), eukaryotic elongation factor 1A, eukaryotic elongation factor 2, translationally controlled tumour protein (TCTP), vimentin and rack1 (Additional file 10: Table S3). All these TOP mRNAs receive the 40 nt spliced leader (SL) RNA sequence at their 5′ ends. The 5′ end of this SL sequence [21] (ACTCATCCCATTTTTGAGTCCGATTTCGATTGTCTAACAG) is pyrimidine-enriched (12 out of the first 15 nucleotides are pyrimidines), although it starts with an adenine and is interrupted by several purines. This suggests that the 5′ end of the spliced-leader may function as a TOP motif in the mTOR-mediated regulation of translation. Our data showed that the translation of *trans*-spliced TOP mRNAs was suppressed upon the inhibition of mTOR: 51 out of the 60 *O. dioica* ribosomal protein mRNAs with translation significantly repressed by mTOR inhibition are *trans*-spliced (Additional file 10: Table S3).

### *Trans*-spliced transcripts dominate the primary translational response to mTOR inhibition

**Fig. 2:**
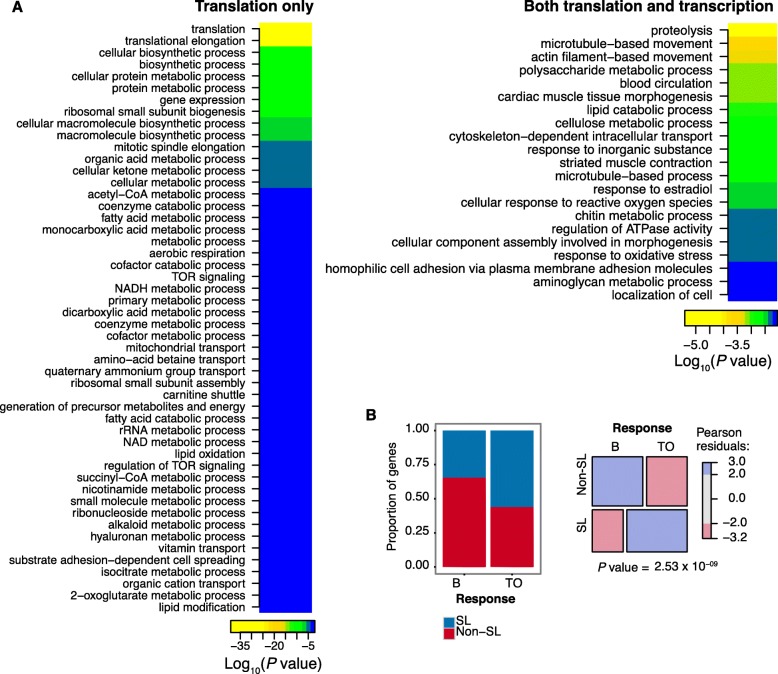
Translational and transcriptional responses to mTOR inhibition. **a** GO analysis of genes with significantly down-regulated translation upon inhibition of mTOR where the response is translational only (left), indicative of a primary response, or both translational and transcriptional (right), indicative of a secondary longer-term response (transcriptional-only comprised only 7 genes and were not analysed further) (**b**) Bar chart (left) shows the proportion of genes with significant down-regulation of translation that are *trans*-spliced (SL) for each response category in (**a**): TO = translation only; B = both translational and transcriptional response. Mosaic plot (right) visualises the Pearson residuals from a corresponding χ^2^ test

*Trans*-splicing of mRNAs is not limited to TOP mRNAs but is associated with 39% of all *O. dioica* genes, a subset that is enriched for a wider array of functions related to growth [5]*.* Of the female-expressed genes that we analysed, 43% are *trans*-spliced. Since the translation of *trans*-spliced TOP mRNAs is mediated via mTOR in *O. dioica,* it opens the possibility that all *trans*-spliced mRNAs are potential targets for growth-dependent translational control. Indeed, we found that 352/762 (46%) of transcripts with suppressed translation upon mTOR inhibition are *trans*-spliced, although this is not significantly more than expected given the frequency of *trans*-splicing. Interestingly, however, we found that mRNAs that were suppressed only at the translation level, and not at the transcription level, were enriched for *trans*-spliced transcripts (56% of mRNAs with translation-only suppression are *trans*-spliced compared to 34% of those with both translational and transcriptional responses to mTOR inhibition, Fisher’s exact test *P-*value = 2.97 × 10^− 9^) (Fig. 2b). This shows that *trans*-spliced transcripts dominate the primary translational response to mTOR inhibition and indicates that non-*trans*-spliced transcripts constitute a longer-term, secondary response involving additional, slower transcriptional adjustment of gene expression. Indeed, GO analysis of these subsets revealed that genes with a transcriptional response to mTOR inhibition were enriched for functions related to proteolysis and muscle contraction (Fig. 2a), the latter being characteristic of genes with transcription down-regulated during growth arrest in *O. dioica* [5].

### Oocyte-stocked mRNAs are *trans*-spliced and translationally dormant

**Fig. 3:**
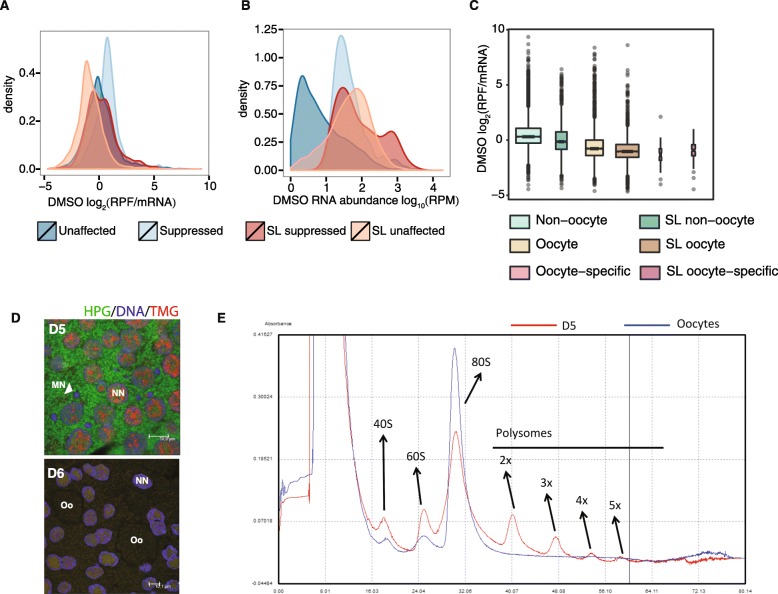
Abundant maternal mRNAs stocked in the oocyte are *trans*-spliced, translationally dormant and resistant to mTOR-inhibition. **a** Distribution of translational efficiencies (ribosome density normalised to mRNA abundance; RPF = ribosome protected fragments) in control (DMSO) animals with transcripts categorised according to the presence of the 5′ spliced leader and their response to mTOR inhibition (suppressed or unaffected). **b** Distribution of mRNA abundances (RPM = reads per million) in control (DMSO) animals with transcripts categorised as in the colour legends under **a** and **b**. **c** Distribution of translational efficiencies in control animals with transcripts categorised by the presence of the 5′ spliced leader and whether or not they are present in oocytes (detected by cap analysis of gene expression (CAGE) or tiling microarray from oocyte samples). **d** The cytoplasm of the coenocyst in day 5 (D5) female gonads, pre-oocyte formation, has a high level of translational activity as indicated by the intensity of green Alexa Fluor® 488 detecting homopropargylglycine (HPG), an amino acid analog that is incorporated during protein synthesis. Nurse nuclei (NN) and meiotic nuclei (MN) are also visible. Oocytes (Oo) that have formed by late day 6 (D6), however, are translationally dormant. Red Alexa Fluor® 568-labelled staining shows the location of mRNAs that have a 5′ trimethylguanosine (TMG) cap, which is present on the spliced leader. DNA was counterstained with blue To-Pro-3 iodide (**e**) RNA from D5 animals have higher levels of polysome occupancy compared to RNA from oocytes, where there are no visible polysome peaks

Surprisingly, given the clear regulation of translation of *trans*-spliced TOP mRNAs, *trans*-spliced genes in female *O. dioica* were, on average, more resistant to mTOR inhibition (mean log_2_ (ΔTE) = − 0.28) than genes that were not *trans*-spliced (mean log_2_ (ΔTE) = − 0.74) (Welch two sample t-test: t = 24.484, df = 14,384, *P*-value < 2.2 × 10^− 16^). *Trans*-spliced transcripts that were not suppressed had significantly lower mean translational efficiencies (mean TE = 1.12), under control conditions, than transcripts that were not *trans*-spliced (mean TE = 2.76) (Welch two sample t-test: t = − 9.6612, df = 11,967, P-value < 2.2 × 10^− 16^) (Fig. 3a) and a significantly higher mRNA abundance (Welch two sample t-test: t = 74.861, df = 12,970, P-value < 2.2 × 10^− 16^) (Fig. 3b). This low level of translation under normal conditions may explain why these transcripts are insensitive to translational suppression via mTOR inhibition. The high abundance but low translation of these mRNAs suggests that they are sequestered: most likely in arrested oocytes, which contain a large fraction of the total RNA pool in this stage of the female *O. dioica* lifecycle and where the majority of transcripts are *trans*-spliced [5]. Fluorescent detection of nascent protein synthesis as well as polysome profiling showed that mRNAs in oocytes are indeed dormant (Fig. 3d,e). Therefore, the majority of weakly-translated, Torin 1-resistant, *trans*-spliced transcripts, likely represent dormant maternal mRNAs stocked in oocytes.

We used both tiling microarray [22] and cap analysis of gene expression (CAGE) [23] data from *O. dioica* oocytes to determine the set of oocyte-stocked mRNAs. As expected, we found that the translational efficiency of oocyte transcripts in control animals was significantly lower than that of non-oocyte transcripts (mean oocyte log_2_ (TE) = − 0.80; mean non-oocyte log_2_ (TE) = 0.45; Welch two-sample t-test: t = − 57.494, df = 14,002, *P*-value < 2.2 × 10^− 16^) (Fig. 3c, Additional file 4: Figure S4), and the effect of Torin 1 was significantly reduced (mean oocyte log_2_ (Δ) = − 0.17; mean non-oocyte log_2_ (Δ) = − 0.97; Welch two-sample t-test: t = 43.54, df = 12,832, P-value < 2.2 × 10^− 16^). Importantly, we found that 80% (4639/5772) of Torin 1-resistant *trans*-spliced transcripts were present in the oocyte. When we removed oocyte transcripts from our analysis, we found that transcripts with suppressed translation upon mTOR inhibition were enriched for those *trans*-spliced with the SL (28.6% of down-regulated genes are *trans*-spliced compared to 17.5% of unaffected genes; Fisher’s exact test P*-*value = 1.26 × 10^− 7^). This is despite excluding most TOP mRNAs, which have transcripts present in the oocyte. We obtained similar results when excluding all transcripts with low levels of translation (DMSO log_2_ (TE) < 1) under control conditions (36.6% of down-regulated genes were *trans*-spliced compared to 22.9% of unaffected genes; Fisher’s exact test P*-*value = 8.4 × 10^− 11^). These results show that while the effect of mTOR inhibition on TOP mRNAs is clear, its full effect on *trans*-spliced mRNAs in general was masked by the abundance of dormant mRNAs in oocytes. Once this is accounted for our results show that mTOR-regulated mRNAs are enriched not only for *trans*-spliced TOP mRNAs but for *trans*-spliced mRNAs in general.

### A TOP motif is not encoded in the genes of mTOR-regulated transcripts

We next wanted to determine whether or not a TOP-like motif is present at the 5′ ends of translation-suppressed transcripts that were not *trans*-spliced. We obtained transcription start sites (TSSs) at bp-resolution in female animals from CAGE data [23] and examined the 5′ sequences of all expressed transcripts. Out of 2772 robustly expressed, non-*trans*-spliced transcripts, only 4 had a canonical TOP motif and only 66 had 5′ pyridine-enrichment comparable to the SL sequence. A more relaxed definition of a TOP-like motif (a stretch of at least 5 pyrimidines starting within 4 nucleotides of a TSS) [9] was also only present at a low frequency (0.076); lower than in mammalian cells (0.16) [9]. We found no significant enrichment of this motif at the 5′ ends of transcripts that had suppressed translation upon mTOR inhibition in *O. dioica*. Since the SL has a stretch of 5 pyrimidines further downstream we also relaxed the definition of the TOP motif further by searching for a stretch of at least 5 pyrimidines within 15 nucleotides of a TSS but still found no significant enrichment in suppressed transcripts. This indicates that these transcripts are indirect targets of translational suppression resulting from a global down-regulation of translation. In further support, a GO term analysis of mTOR-regulated transcripts lacking a spliced leader revealed an enrichment of functions related to autophagy (proteolysis) and lipid catabolism whereas those that were *trans*-spliced were enriched for known TOP mRNA functions related to protein synthesis. These results provide further evidence that the spliced leader supplies the TOP-like motif necessary for mTOR regulation and that the primary translational response to mTOR-inhibition is dominated by the selective suppression of *trans*-spliced mRNAs.

### *Trans*-spliced transcripts in *C. elegans* are under growth-dependent translational control

We next sought to identify *trans*-spliced TOP mRNAs that are under mTOR regulation in another metazoan species. *C. elegans trans*-splices 70% of its mRNAs to one of two pyrimidine-enriched spliced leaders [4, 24]. SL1 is associated with monocistrons and the first gene in an operon and SL2 is associated with downstream operon genes. Included amongst these are all but one ribosomal protein gene (TOP mRNAs), which are mostly *trans*-spliced with SL1 [5]. A genome-wide study of translation during L1 diapause exit identified ribosomal protein mRNAs as transcripts with the highest translational up-regulation [25]. While no mention of the association of these transcripts with *trans*-splicing was made in this study, the data nevertheless clearly showed that *trans*-spliced ribosomal protein (TOP) mRNAs were targets of nutrient-dependent translational-control. Furthermore, a recent study showed that t*rans*-splicing in *C. elegans* enhances translational efficiency [26].

In order to establish whether or not a relationship exists between the presence of SL1 and/or SL2 at the 5′ end of an mRNA and its translational control during recovery from growth arrest, we re-analysed existing ribosome profiling and mRNA-seq data from L1 diapause exit [25] together with existing data mapping *trans*-splice sites genome-wide in *C. elegans* [24]. We used a total of 10,362 genes that could be tested for differential translational regulation and assigned a *trans*-splicing category with high confidence. Amongst these, we found a strong relationship between the presence of a 5′ spliced leader and translational control during L1 diapause exit in response to food availability (χ^2^ = 711.45, df = 4, *P* value < 2.2 × 10^− 16^) (Additional file 5: Figure S5). Amongst transcripts with up-regulated translation, 54% (786/1460) are *trans*-spliced to SL1 and 18% (260/1460) are *trans*-spliced to SL2, while 414 (28%) lack a 5′ spliced leader (Additional file 5: Figure S5). This constitutes an enrichment of *trans*-spliced transcripts compared to unaffected transcripts (56% of which lack a 5′ spliced leader). These results show that *trans*-spliced TOP mRNAs in *C. elegans* are also under nutrient-dependent translational control, indicating that the spliced leaders in *C. elegans* may be targets of mTOR.

### Exit from growth arrest in *O. dioica* is not dependent on mTOR

Having established that *trans*-spliced transcripts in female *O. dioica* are targets of mTOR-regulated translational control and that translation of *trans*-spliced TOP mRNAs are up-regulated during recovery from growth arrest in *C. elegans*, we next wanted to assess the translational regulation of *trans*-spliced transcripts during recovery from growth arrest in *O. dioica*.

We previously proposed that translational control, rather than transcriptional control, may up-regulate *trans*-spliced growth-related genes during recovery [5]. To test this we performed ribosome profiling followed by deep sequencing, together with total RNA sequencing, on *O. dioica* during growth arrest (stasis: animals were collected on day 7, one day beyond their normal 6-day lifespan) and recovery from growth arrest (release into normal animal density).

Sequencing generated 27.0 M (stasis) and 38.6 M (release) total RNA exon-mapped reads and 1.8 M (stasis) and 1.5 M (release) RPF exon-mapped reads, across two biological replicates.

We detected 1601 genes with significantly up-regulated transcription and 638 with significantly down-regulated transcription during release from stasis. Consistent with our previous observations [5], genes that were transcriptionally up-regulated were enriched for muscle-related GO terms and *trans*-splicing was under-represented in this set (Additional file 6: Figure S6).

**Fig. 4:**
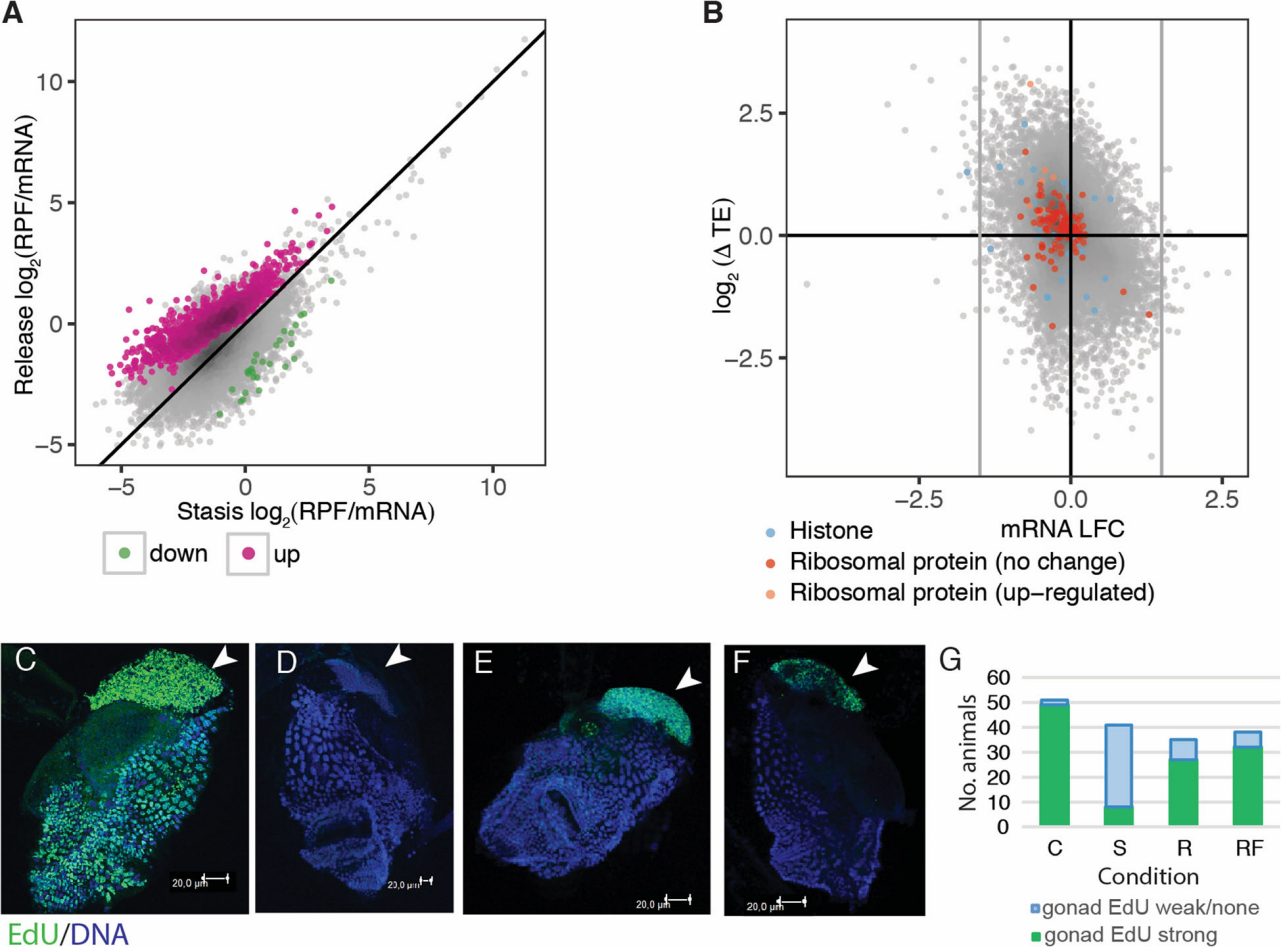
The growth arrest recovery translatome of *O. dioica*. Animals were cultured under dense conditions before being released by dilution in the presence of food. **a** Median translational efficiency (RPF/mRNA = ribosome protected fragment density/mRNA density) of mRNAs from 2 replicates for growth arrested and released animals with transcripts identified as having significantly up- or down-regulated translation highlighted. **b** Degree of change in translational efficiency upon release from stasis (y-axis) against log fold change in mRNA abundance (x-axis) with known mTOR-independent (histone mRNAs) and mTOR target (ribosomal protein mRNAs) gene categories highlighted. The majority of mTOR targets are not up-regulated upon release from stasis. **c**-**g** EdU incorporation (DNA replication: green) was restored in the germline (top in each image, as indicated by arrows) 12 h after animals were released from the crowded conditions of growth arrest independent of food supply confirming that a change in density rather than increased food availability is the primary trigger for exiting a growth-arrested state in *O. dioica* (DNA was counterstained with blue To-Pro-3 iodide). **c** normal day 3; **d** growth arrest; **e** release without food; **f** release with food; **g** number of animals showing extensive DNA synthesis under all four conditions (C = normal day 3 control; S = stasis; R = release without food; RF = release with food)

We then analysed differential translational efficiency and found 1382 genes with significantly up-regulated translational efficiency upon release from stasis and only 28 significantly down-regulated (Fig. 4). Surprisingly, we found that only 8/129 ribosomal protein mRNAs were up-regulated (Fig. 4b). *Trans*-spliced transcripts were not over-represented in the set of up-regulated genes and the mean change in translational efficiency was not significantly different between *trans*-spliced and non-*trans*-spliced transcripts (t-test: t = − 0.32652, df = 13,368, *P* value = 0.744). GO terms that were over-represented in the set of genes with up-regulated translational efficiencies included terms related to muscle contraction, hormone regulation and the cell cycle (Additional file 7: Figure S7), rather than terms typical of the mTOR-dependent translatome we identified in our mTOR-inhibition experiments.

These results show that up-regulation of nutrient-dependent growth-related genes (genes regulated by mTOR) is not the initial response to release from growth arrest in *O. dioica.* Supporting this, replication tracing by EdU incorporation showed that endocycling, which is suppressed during growth arrest, resumed in released animals regardless of whether or not food was available (Fig. 4c-g).

## Discussion

We have shown that *trans*-splicing of a spliced leader sequence to the 5′ ends of mRNAs is associated with growth-dependent translational control in two different metazoans.

**Fig. 5:**
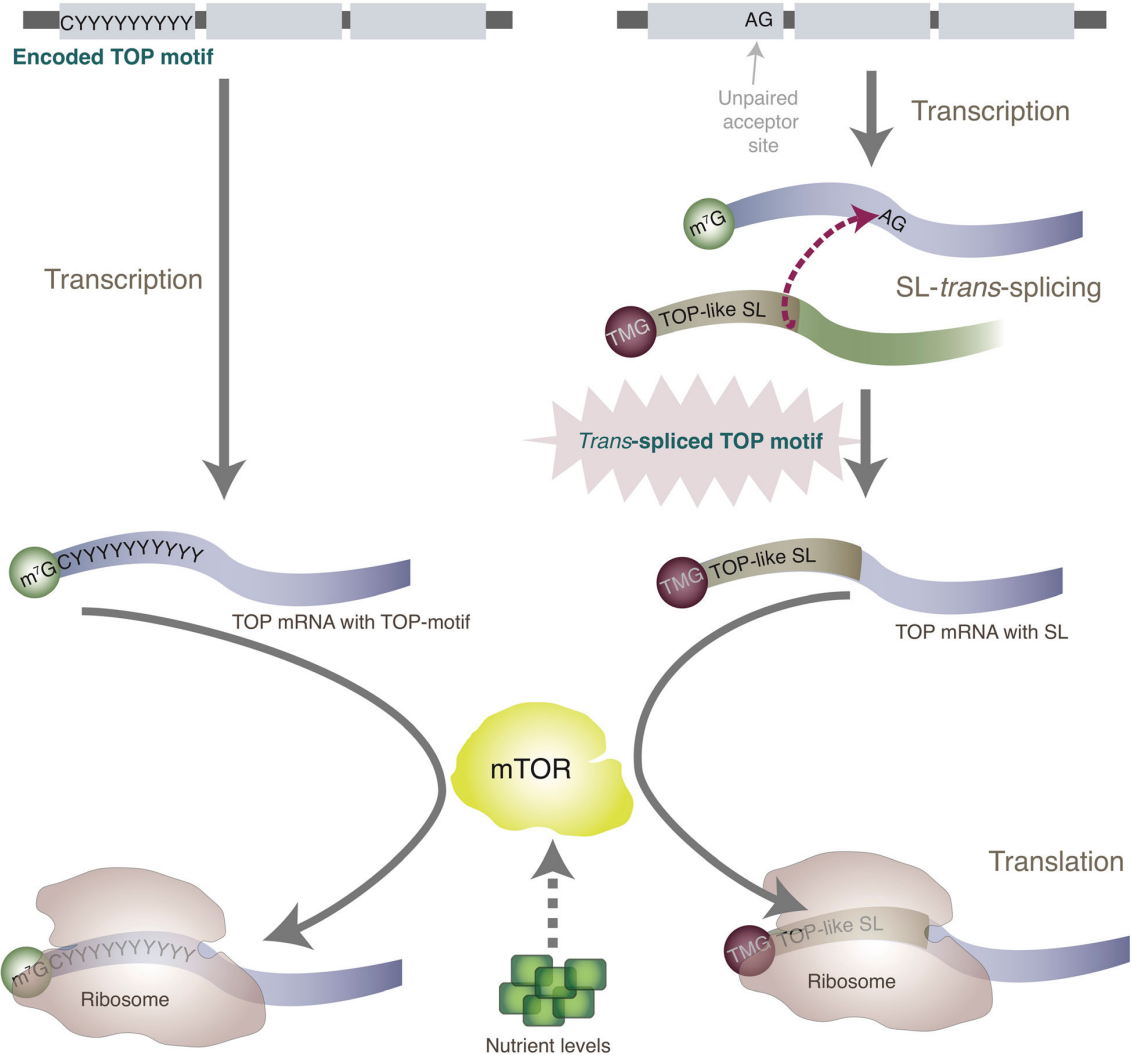
Translational control of TOP mRNAs. Schematic shows our working model for the translational control of TOP mRNAs in animal genomes where a TOP motif is encoded at gene loci (left) and in animals where a TOP-like sequence is added post-transcriptionally via *trans*-splicing (right). The nutrient-dependent translation of classical TOP mRNAs (predominantly ribosomal proteins) in mammalian cells is controlled via mTOR signalling and requires a 5′ Terminal Oligopyrimidine Tract (TOP motif). In *O. dioica* and *C. elegans,* the TOP motif is not encoded in the 5′ UTRs of many such genes, but instead, these mRNAs are *trans*-spliced with a spliced leader (SL) that has a pyrimidine-enriched 5′ end and a 5′ trimethylguanosine (TMG) cap. We showed that the translation of these *trans*-spliced TOP mRNAs was mediated by mTOR in *O. dioica* suggesting the potential for remarkable evolutionary innovation, and flexibility, in the regulation of growth in these animals

Inhibiting mTOR in *O. dioica* revealed a typical mTOR-dependent translatome with classical TOP mRNAs as primary targets. These TOP mRNAs do not contain the highly conserved TOP motif, as in other species, but are instead *trans*-spliced with a leader sequence resembling the pyrimidine-enriched TOP-like motif. Our results support a working model where the SL performs the same role as a TOP motif in permitting nutrient-dependent translational control of mRNAs via mTOR (Fig. 5). Nutrient-induced recovery from L1 arrest in *C. elegans* involves the translational up-regulation of mTOR-regulated transcripts that are also *trans*-spliced.

The potential survival advantage of an association between mTOR and the spliced leader may be a key driving force in the evolution (or retention) of *trans*-splicing. The *trans*-splicing of translational control motifs may also permit the rapid evolution of new targets for mTOR-regulation, given that all that is required is an unpaired acceptor site for *trans*-splicing of the spliced leader, rather than the evolution of the complete TOP motif. In addition, the regulation of *trans*-splicing itself allows for the switching on or off of mTOR targets at different stages of the life cycle. This may be achieved through the use of alternative start sites that either include or exclude the *trans*-splice site. Supporting this, a male-specific promoter motif recently identified in *O. dioica* [23] causes the exclusion of *trans*-splice sites, and consequently the lack of a spliced leader on resulting transcripts, in many cases.

Given the strong association of *trans*-splicing with maternal mRNA, mTOR regulation via the spliced leader may be an underlying mechanism for the adjustment of egg numbers according to nutrient levels in *O. dioica* [11]. This implicates *trans*-splicing as a possibly important, molecular-level mediator of population levels in this abundant zooplankton.

Our results suggest that the initial trigger for an exit from growth arrest in *O. dioica* is not primarily an increase in nutrient levels. Instead, the animals appear to be responding more to the return to normal animal densities. This indicates that growth arrest in *O. dioica* exhibits some similarity to dauer arrest in *C. elegans*, which relies on the relative amounts of a dauer pheromone and a “food signal” (rather than L1 arrest, which is induced by starvation). The search for pheromones in *O. dioica* would shed further light on this.

Our observations of animals that were released from growth arrest with and without food indicate that nutrition is, however, critical for the maturation (gametogenesis) of *O. dioica.* Animals that were released with food matured, spawned and died normally after 3 days whereas animals without food died after 3 days with under-developed gonads and did not spawn (data not shown). This is in agreement with our findings that the translation of *trans*-spliced TOP mRNAs is regulated by mTOR during oogenesis (day 6), as well as previous findings that egg numbers are dependent on nutrient levels [11]: without food animals do not have sufficient resources to produce oocytes so they die without spawning. Interestingly, animals that matured after release with food were predominantly females (81.7% females vs 18.2% males; average from 3 experiments where animals were released in the presence of food). The reason for this is at present, unclear.

Despite sharing a common 5′ end motif in the SL, not all *trans*-spliced transcripts were affected by mTOR inhibition. We showed that many *trans*-spliced TOP mRNAs were stocked in oocytes and were translationally dormant, which largely accounts for this resistance. Interestingly, however, we also found a small subset of 21 actively translated *trans*-spliced transcripts in *O. dioica* that were resistant to Torin 1. Furthermore, in *C. elegans*, while there was an enrichment of *trans*-spliced transcripts in the set of transcripts with up-regulated translation during L1 diapause exit, a large fraction (59%; 753/1282) of transcripts with down-regulated translation are also *trans*-spliced (with SL1). This may indicate cell-type specific sequestering of a subset of SL1 transcripts upon recovery. The translation of several known TOP mRNAs is regulated in a cell-type specific manner [27] and some, including poly(A) binding protein (PABP), contain downstream sequence motifs that can override the TOP motif [18]. It is possible, therefore, that mRNAs share regulatory motifs in the SL sequence but respond differently to mTOR depending on their cell-type and sequence contexts. Additional tissue- and developmental stage-specific SL-CAGE datasets would be beneficial to explore this further.

## Conclusion

Here we showed that the translation of *trans*-spliced TOP mRNAs, which determines the levels of protein synthesis in the cell, and therefore growth, was regulated by mTOR in *O. dioica*. These mRNAs were under nutrient-dependent translational control during a developmental stage in which the majority of transcriptional output is allocated towards egg production. Our results are consistent with a working model where *trans*-splicing could provide an evolutionary advantage in the ability to rapidly alter resource allocation during vitellogenesis according to nutritional cues from the environment. We also showed that initial recovery from growth arrest in *O. dioica* was not triggered by increased nutrient levels and, accordingly, that the translational response was not dominated by *trans*-spliced transcripts. Further work on a range of additional metazoans would provide further insight into the evolution of *trans*-splicing and its role in translational control during oogenesis. Additional mechanistic data are also required to show unequivocally that the 5′ end of the SL is the primary target of mTOR regulation in these species.

## Methods

### Materials

Reagents were obtained from the following sources: antibodies to phospho-4E-BP1 (Thr37/46) from Cell Signalling (#2855); H3 antibodies from Abcam (#ab1791); anti-rabbit IgG secondary antibodies from KPL (#074–1506); Lambda Protein Phosphatase (Lambda PP) from New England Biolabs (#P0753S); ARTseqTM kit from Epicentre (#RPHMR12126); RNaqueous kit from Ambion (#AM1912); Torin 1 from Tocris (#4247); protease inhibitor cocktail from Sigma-Aldrich (P2714-1BTL); Halt™ Phosphatase Inhibitor Cocktail from Thermo Scientific (#78420); precast gels from BioRad (#4566033 and #4565013); To-Pro-3 iodide from Molecular Probes (#T3605); VECTASHIELD® from Vector Laboratories (#H1000); EdU from Thermo Fisher Catalog (#A10044); Click-iT™ HPG Alexa Fluor™ 488 Protein Synthesis Assay Kit from Thermo Fisher (#C10428); TMG-cap antibodies from Santa Cruz (#sc-32,724); Zymo RNA Clean & Concentrator-25 kit (#R1018); Ribo-Zero (#MRZG12324); Clarity Western ECL Substrate from BioRad (#170–5060).

### Culture of animals

Animals were cultured as previously described [28].

### Collection of day 6 females for ribosome profiling

At day 6, 80 female animals were transferred to 3 L beakers of seawater containing regular algal food concentrations and treated with either 1 μM of Torin 1 (treatment) or DMSO (vehicle control). Animals were collected after 1.5 h and examined under a microscope to confirm they were female and to remove their houses. The majority of collected animals were washed in PBS and frozen in liquid nitrogen for ribosome profiling. A small sample of animals collected for each replicate was added to 2x Laemmli buffer (4% SDS, 10% 2-mercaptoethanol, 20% glycerol, 0.004% bromophenol blue, 0.125 M Tris HCl) in a 1:1 ratio and frozen in liquid nitrogen for Western blot to confirm the absence of phosphorylated 4E-BP1 upon Torin 1 treatment.

### Collection of animals during recovery from growth arrest for ribosome profiling

Animals were collected as previously described [5] at day 7 of growth arrest and 30 min following release, with 700–1000 animals collected per sample.

### Preparation of RPF libraries

We used the ARTseq^TM^ kit and followed the kit protocol with minor modifications. Briefly, animal samples were thawed in lysis buffer, omitting cycloheximide to avoid a 5′ end bias. Lysates were clarified by centrifugation for 10 min at 20,000 x g at 4 °C. Amount of ARTseq nuclease was optimized, following steps in the ARTseq kit protocol, resulting in 2 units used to digest RNA. Monosomes were purified using MicroSpin S-400 columns and centrifugation for 2 min at 600 x g. Ribosome protected RNA fragments (RPFs) were purified using Zymo RNA Clean & Concentrator-25 kit. rRNA was removed using Ribo-Zero and RPFs PAGE purified, eluted and precipitated and resuspended in nuclease-free water and libraries prepared for sequencing following the kit protocol. Libraries were prepared in three biological replicates.

### Preparation of total RNA libraries for ribosome profiling

Total RNA was extracted from a subset of animals from each sample (15 day 6 females; 160–250 stasis/release) using the RNEasy kit and rRNA removed using Ribo-Zero. RNA was heat fragmented prior to library preparation using the ARTseq kit protocol and reagents. Libraries were prepared in three (day 6 females) or two (stasis/release) biological replicates.

### Western blots

Samples were lysed with 2x Laemmli buffer (4% SDS, 10% 2-mercaptoethanol, 20% glycerol, 0.004% bromophenol blue, 0.125 M Tris HCl) in a 1:1 ratio followed by mechanical disruption using disposable pestles or vortex. Mixtures were heated at 95 °C for 5 min followed by 5 min centrifugation. Whole cell extracts were fractionated by SDS-PAGE using precast stain free gels. Proteins were transferred to a 0.45 μm nitrocellulose membrane (GE Healthcare Life Science) and blocked in 5% BSA diluted in 1x TBST (Tris Buffer Saline with 0.1% Tween-20) for 1 h at room temperature. Primary and secondary antibodies were diluted in blocking buffer (1:1000 and 1:2000, respectively) and membranes developed with the Clarity Western ECL Substrate (BioRad), according to the manufacturer’s recommendations.

### RT-qPCR validation

RNA was extracted from the same subset of the samples used for polysome profiling (2 biological replicates for DMSO-treated D6 females and 2 biological replicates for Torin1-treated D6 females) using a phenol-chloroform extraction [29]. Briefly, 1 volume of acid phenol:chloroform:isoamyl alcohol (pH 4.5) was added and incubated at 65 °C for 5 min. Samples were centrifuged at 15,000 x g at room temperature for 5 min, followed by transfer of the aqueous phase and a second addition of 1 volume acid phenol:chloroform:isoamyl alcohol (pH 4.5) and centrifugation. Aqueous phase was transferred and 1 volume chloroform-IAA (24:1) added. After separation of phases by centrifugation, the RNA was precipitated by addition of 1 volume isopropanol, 0.1 volume NaOAc and glycogen while incubated at − 20 °C overnight. RNA was pelleted by centrifugation, washed once in 80% ethanol, and redissolved in water. Genomic DNA was removed by Turbo DNase treatment and cleaned up with Zymo RNA Clean & Concentrator-25 kit (#R1018).

cDNA was synthesized using 400 ng of RNA per reaction, random hexamers and SOLIScript reverse transcriptase (following manufacturer’s instructions, #06–35-00050). cDNA samples were diluted as necessary for qPCR reactions. The RT-qPCR was performed in 20 μl reactions using SsoAdvanced Universal SYBR®Green Supermix from BioRad (#172–5274). Primers were designed (Additional file 11: Table S4), commercially synthesized by Sigma Aldrich and used at a final concentration of 500 nM each (forward and reverse). Elongation factor 1-delta was used as a normalization control, using quadruplicate technical replicates for all reactions. Reactions were run on the CFX96 (BioRad) following the program; 95 °C for 5 min, 40 cycles of 95 °C for 15 s, 58 °C for 20 s and 72 °C for 20 s, and one cycle at 72 °C for 5 min. Relative expression of genes tested was measured using the reference gene and delta-Ct values. DMSO and Torin 1 results were compared to each other for each gene tested using a Welch two-sample t-test.

### Ribosome profiling sequencing data analysis

Libraries were sequenced on two Illumina rapid flow cells at the Genomics Core Facility at the Norwegian University of Science and Technology (NTNU). We clipped adapter sequences from reads before mapping to *O. dioica* rRNA and tRNA sequences using Genoscope annotations and Bowtie2. Remaining reads were mapped to the *O. dioica* reference genome using TopHat and Genoscope gene model annotations as a guide. We used the R package *Rsubread* to calculate read counts for each protein-coding gene and the R package *babel* [30] for differential translational efficiency analysis. Genes with fewer than 100 reads across all samples were excluded from further analysis. This gave us a final set of 14,574 expressed genes. We used previously published CAGE data mapping SL-*trans*-splice sites genome-wide [5] and classed a gene as SL *trans*-spliced if there was a *trans*-splice site within the gene body or within a 500 bp upstream region, if it was supported by > 1 tag count and if it had an ‘AG’ acceptor site motif immediately upstream. Trends in translational efficiency changes were analysed by normalising all read counts to reads per million (RPM) for each library, averaging replicates and normalising RPF RPM to total RNA RPM for each gene model.

### Cap analysis of gene expression (CAGE)

We extracted locations of TSSs in day 6 females from an existing CAGE data set [23]. We used the sequence immediately downstream of TSSs to search for TOP and TOP-like motifs.

### Defining oocyte transcripts

We used tiling array data [22] and CAGE [23] data generated from *O. dioica* oocytes to define oocyte-stocked mRNA transcripts.

Any gene with an average probe intensity > 0 in the tiling array data or associated with a dominant CAGE TSS ≥ 1 tpm within the gene body or 500 bp upstream region were classed as oocyte transcripts.

### Gene ontology (GO) analysis

We used *O. dioica* GO annotations [22] and the Bioconductor *GOstats* package in R to compute hypergeometric *P*-values for over-representation of GO terms.

### Collection of immature day 6 females for polysome profiling

At day 5, female animals were separated from males, by visual inspection under a microscope, and cultured under normal conditions to day 6. At day 6, 80 immature animals were transferred to 3 L beakers of seawater containing regular algal food concentrations and treated with either 1 μM of Torin 1 (treatment) or DMSO (vehicle control). Animals were collected after 1.5 h and examined under a microscope to remove their houses. Collected animals were washed in PBS and frozen in liquid nitrogen.

### Polysome profiling

Samples were lysed with mammalian lysis buffer (200 μl 5x Mammalian polysome buffer, 100 μl 10% Triton X-100, 10 μl 100 mM DTT, 10 μl Dnase I (1 U/μl), 678 μl Nuclease-free water) supplemented with 2 μl cyclohexamide. Lysates were placed on a sucrose gradient (15–45%) and centrifuged in a SW41 rotor at 36,000 rpm for 2 h at 4 °C. Gradients were fractionated using the Piston Gradient Fractionator (Biocomp) with in-line OD260 in-line absorbance monitoring to create the profiles.

### EdU incorporation assay

Animals for growth arrest were maintained as previously. Animals were released after 7 days of food–restricted, dense culture conditions by manual transfer into clean sea water at standard culture densities, with or without standard algal strain mixture. After 12 h, EdU (5-ethynyl-2′-deoxyuridine)-labeling assay was done according to the manual on 50 animals per sample with incubation time 30 min. Fixation in 4% PFA was followed by Click-iT reaction with Alexa 488. DNA was counterstained with 1 μM To-Pro-3 iodide and mounted in VECTASHIELD®. Samples were analyzed by confocal microscopy using a Leica TCS laser scanning confocal microscope and Leica (LAS AF v2.3) software.

### ClickIT translational assay

Click-iT™ HPG Alexa Fluor™ 488 Protein Synthesis Assay Kit was used with minor modifications. Animals were incubated in 50 μM Click-iT® HPG reagent in sea water for 1 h to label newly translated proteins. After fixation in 4% PFA, standard immunofluorescence staining [14] was performed using mouse TMG-cap antibody. After secondary antibody washes, Click-iT™ substrate detection was performed, according to the manual, DNA was counterstained with 1 μM To-Pro-3 iodide and samples were mounted in VECTASHIELD® for confocal microscopy.

### Analysis of *C. elegans trans*-splicing and ribosome profiling data

We defined *trans*-spliced genes using existing data [24]. Genes with SL2 reads comprising ≤ 25% of total *trans*-spliced reads and a binomial exact *P* value for this percentage < 0.05 were classed as SL1. Genes with SL2 comprising ≥ 75% of reads (P value < 0.05) were classed as SL2. We classed remaining genes as “mixed” if percentages fell between these thresholds or if there were two or more *trans*-splice sites using both spliced leaders, or “unknown” if *P* values were ≥ 0.05.

Remaining genes (using WS207 annotations of protein coding transcripts following [24]) were classed as non-SL. WS207 gene sequence names were converted to WS230 and mapped to Wormbase protein IDs version WS235 in order to map to gene annotations used in previously generated ribosome profiling data [25]. We classed genes as translationally up-regulated upon exit from L1 diapause if there was a RPF fold change > 1 and an adjusted *P* value < 0.001. Genes were classed as down-regulated if there was a RPF fold change < 1 and an adjusted *P* value < 0.001. All other genes were classed as unaffected.

## Supplementary information

**Additional file 1: Figure S1.** Response to the mTOR inhibitor Torin 1 in *O. dioica*. (A-C) Female animals were exposed to DMSO (vehicle control) and different concentrations (50 nM, 250 nM, 1250 nM and 5000 nM) of the mTOR inhibitor, Torin 1, and 4E-BP1 phosphorylation levels were assayed. Histone H3 was used as a reference loading control. Annotated summary is shown (A) as well as full blots for 4E-BP1 phosphorylation (B) and H3 (C). An annotated full blot (D) shows the presence of phosphorylated 4E-BP1 in the presence of DMSO and its absence upon mTOR inhibition with 1 μM Torin 1 in samples taken during three animal collections for ribosome profiling (see also Fig. 1a). A sample treated with Lambda-phosphatase is shown in (E) demonstrating the specificity of the antibody against the phosphorylated form of 4E-BP1 in *O. dioica*.

**Additional file 2: Figure S2.** Global translational response to treatment with the mTOR inhibitor Torin 1. Polysome profiles from two replicates of Torin 1 treated (T1 and T2) and DMSO control (D1 and D2) day 6 animals confirmed a down-regulation of translation in treated animals as indicated by reduced polysome peaks.

**Additional file 3: Figure S3.** Conserved functions in the targets of mTOR-dependent translational control in *O. dioica*. GO terms and *p*-values from a gene ontology (GO) analysis of genes with transcripts that had significantly down-regulated translation upon mTOR inhibition with Torin 1.

**Additional file 4: Figure S4.** Oocyte transcripts are *trans*-spliced and translationally dormant. Changes in translational efficiency in response to Torin 1 (y-axis) against mRNA abundances (RPM = reads per million) in control animals (x-axis) with transcripts categorised as indicated in the legend.

**Additional file 5: Figure S5.** Translational control during nutrient-dependent recovery from growth arrest is associated with the presence of a 5′ spliced leader in *C. elegans*. (A) Proportion of genes *trans*-spliced to SL1 or SL2 or without a spliced leader that have translation up- or down-regulated (or no translational response) upon release from L1 diapause in response to food availability. (B) Mosaic plot shows Pearson residuals from a Chi-square test using genes categorised as in (A).

**Additional file 6: Figure S6.** Transcriptional response during recovery from growth arrest in *O. dioica*. Genes with significantly up-regulated transcription during recovery from growth arrest were enriched for non-*trans*-spliced transcripts (A) and GO terms related to lipid metabolism, muscle contraction and proteolysis (B).

**Additional file 7: Figure S7.** Translational response during recovery from growth arrest in *O. dioica*. GO terms enriched in genes with significantly up-regulated translation during recovery from growth arrest.

**Additional file 8: Table S1.** Mapping statistics of ribosome protected fragment and total RNA libraries.

**Additional file 9: Table S2.** Translational efficiencies in control and treated animals for all tested genes.

**Additional file 10: Table S3.** Translational control of *O. dioica* mRNAs associated with genes that are orthologous to human genes encoding TOP mRNAs.

**Additional file 11: Table S4.** Primers used for RT-qPCR validation.

## Data Availability

High-throughput sequencing data have been deposited at the NCBI Gene Expression Omnibus (http://www.ncbi.nlm.nih.gov/geo/) under accession numbers GSE78807 and GSE115265. A preprint version of this manuscript can be accessed at the following link: https://www.biorxiv.org/content/early/2018/06/22/353979.

## Abbreviations

4E-BP1: eukaryotic translation initiation factor 4E binding protein 1
CAGE: Cap analysis of gene expression
DMSO: Dimethyl sulfoxide
EdU: 5-ethynyl-2′-deoxyuridine
eIF4E: eukaryotic translation initiation factor 4E
eIF4G: eukaryotic translation initiation factor G
GO: Gene ontology
MEF: mouse embryonic fibroblast
mTOR: mammalian target of rapamycin
mTORC1: mammalian target of rapamycin complex 1
PABP: poly(A) binding protein
qRT-PCR: quantitatitve reverse transcription polymerase chain reaction
RPF: ribosome protected RNA fragments
RPM: reads per million
SL: Spliced leader
TCTP: translationally controlled tumour protein
TE: translational efficiency
TOP: Terminal OligoPyrimidine
tpm: tags per million
TSS: transcription start site
