# Supplementary figures and images for: Trans-splicing of mRNAs links gene transcription to translational control regulated by mTOR
Source: BMC Genomics. 2019 Nov 29;20:908. doi: 10.1186/s12864-019-6277-x (PMC6883708; doi:10.1186/s12864-019-6277-x)

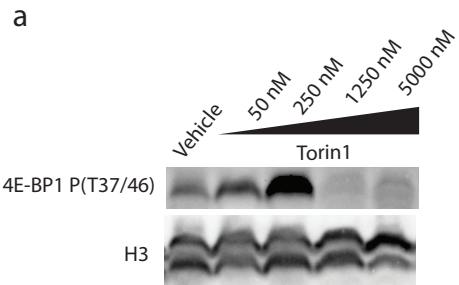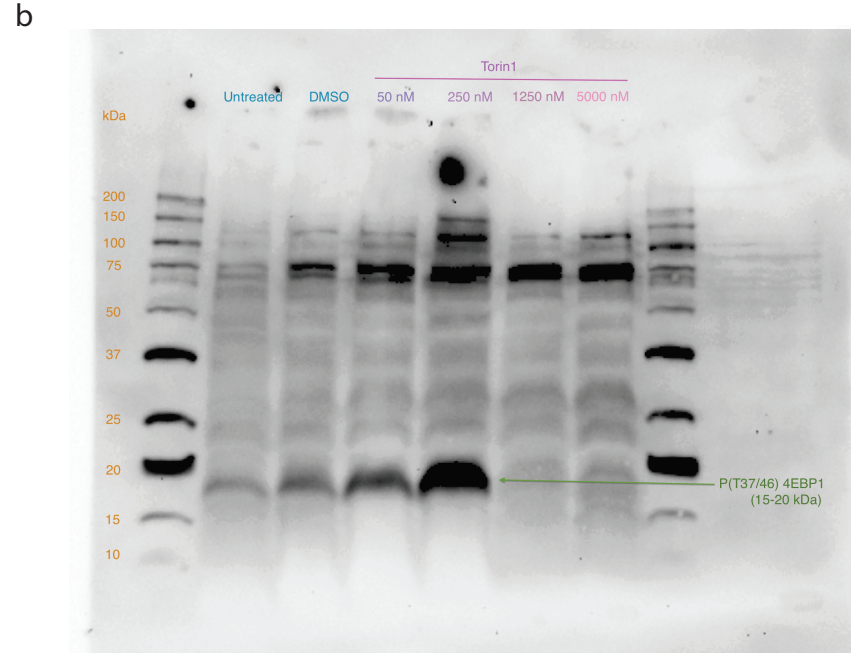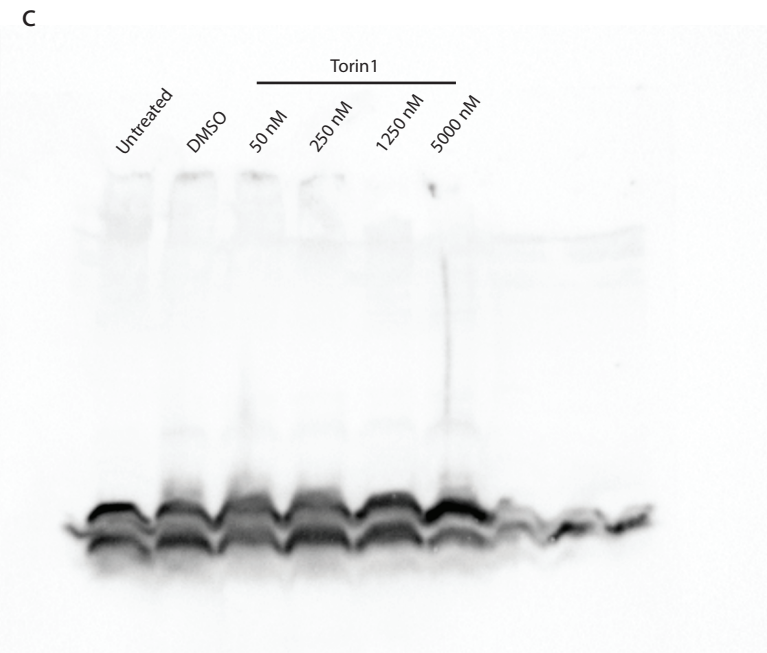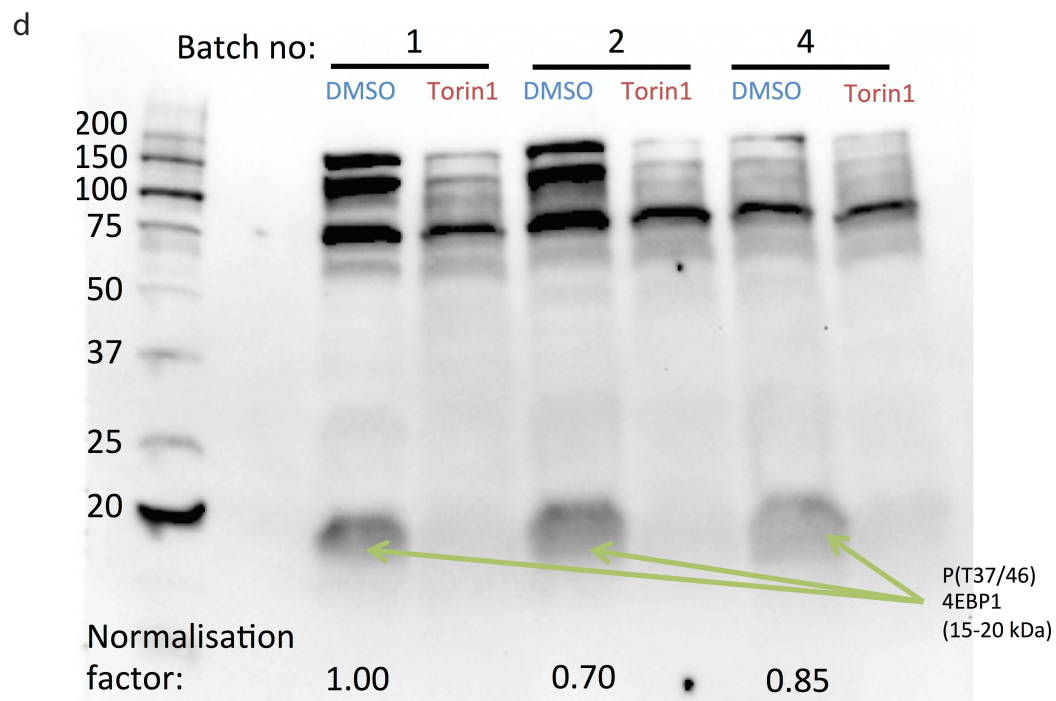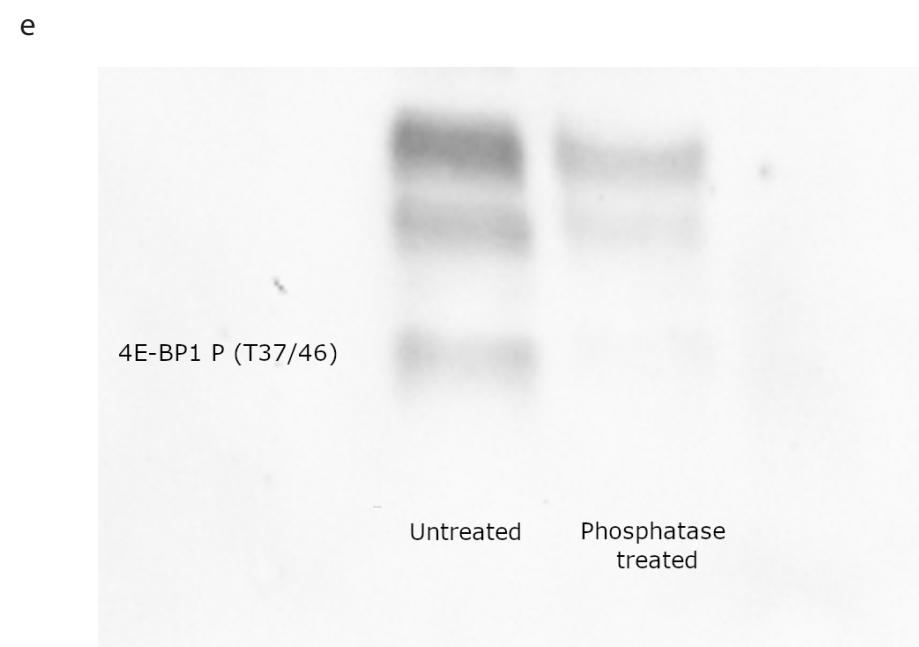

Supplement: Supplementary file 1 — Additional file 1: Figure S1. Response to the mTOR inhibitor Torin 1 in O. dioica. (A-C) Female animals were exposed to DMSO (vehicle control) and different concentrations (50 nM, 250 nM, 1250 nM and 5000 nM) of the mTOR inhibitor, Torin 1, and 4E-BP1 phosphorylation levels were assayed. Histone H3 was used as a reference loading control. Annotated summary is shown (A) as well as full blots for 4E-BP1 phosphorylation (B) and H3 (C). An annotated full blot (D) shows the presence of phosphorylated 4E-BP1 in the presence of DMSO and its absence upon mTOR inhibition with 1 μM Torin 1 in samples taken during three animal collections for ribosome profiling (see also Fig. 1a). A sample treated with Lambda-phosphatase is shown in (E) demonstrating the specificity of the antibody against the phosphorylated form of 4E-BP1 in O. dioica. [file 12864_2019_6277_MOESM1_ESM.pdf]

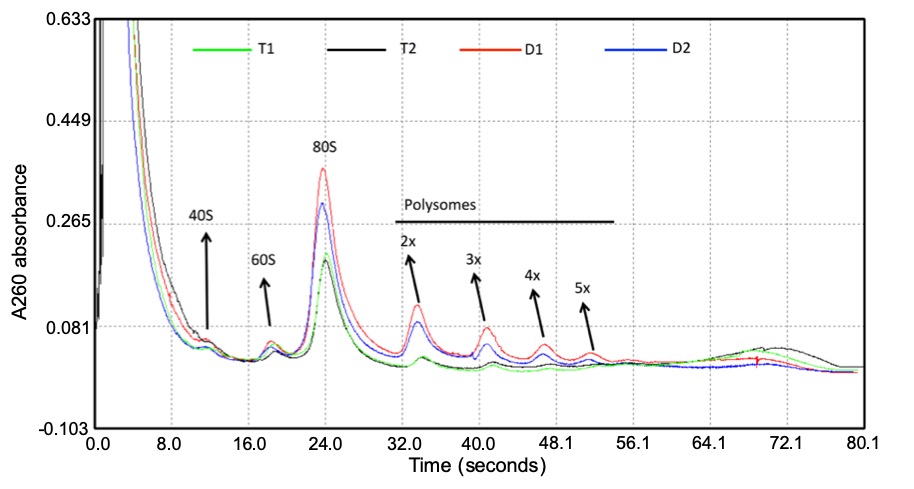

Supplement: Supplementary file 2 — Additional file 2: Figure S2. Global translational response to treatment with the mTOR inhibitor Torin 1. Polysome profiles from two replicates of Torin 1 treated (T1 and T2) and DMSO control (D1 and D2) day 6 animals confirmed a down-regulation of translation in treated animals as indicated by reduced polysome peaks. [file 12864_2019_6277_MOESM2_ESM.jpg]

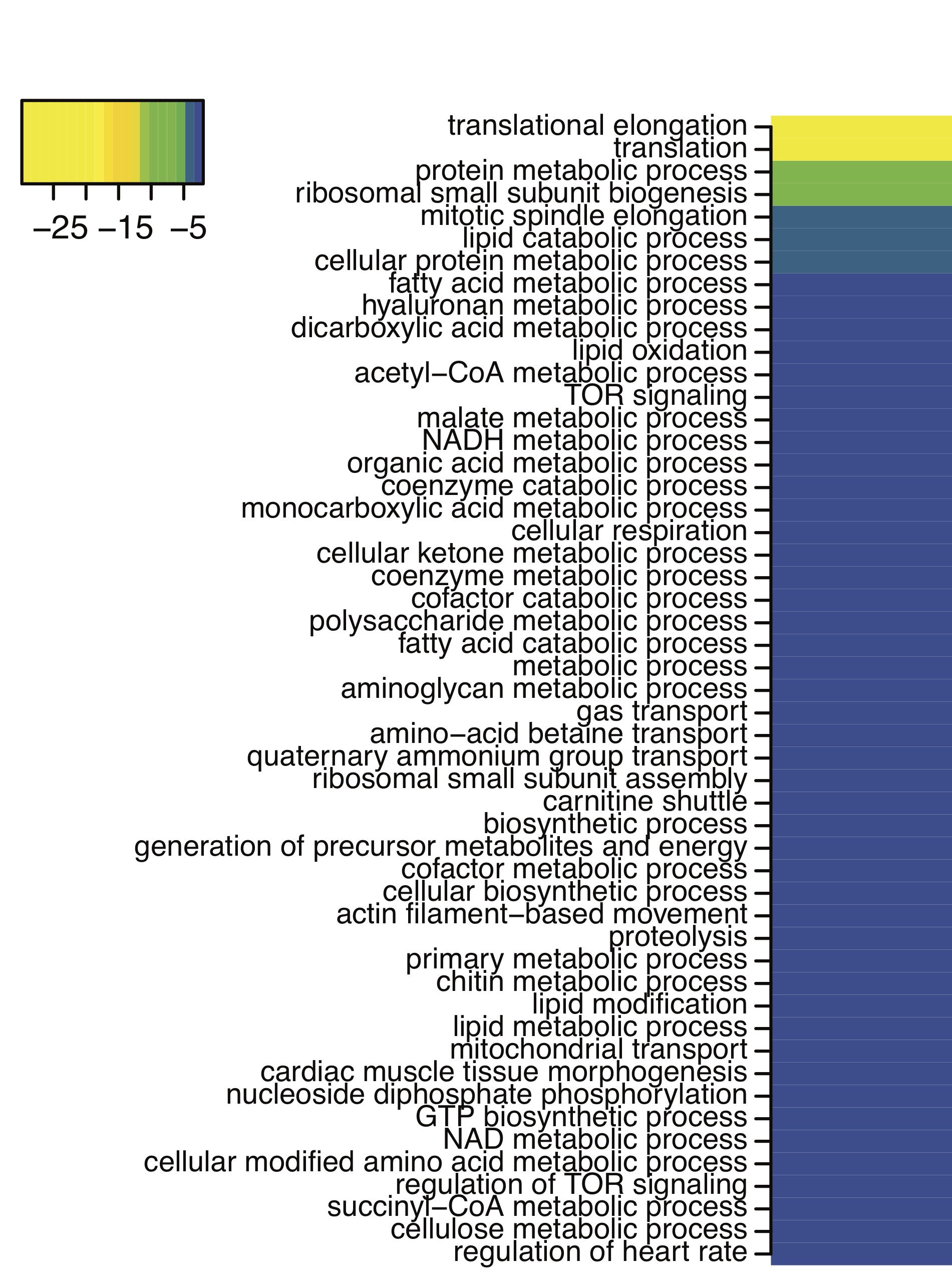

Supplement: Supplementary file 3 — Additional file 3: Figure S3. Conserved functions in the targets of mTOR-dependent translational control in O. dioica. GO terms and p-values from a gene ontology (GO) analysis of genes with transcripts that had significantly down-regulated translation upon mTOR inhibition with Torin 1. [file 12864_2019_6277_MOESM3_ESM.jpg]

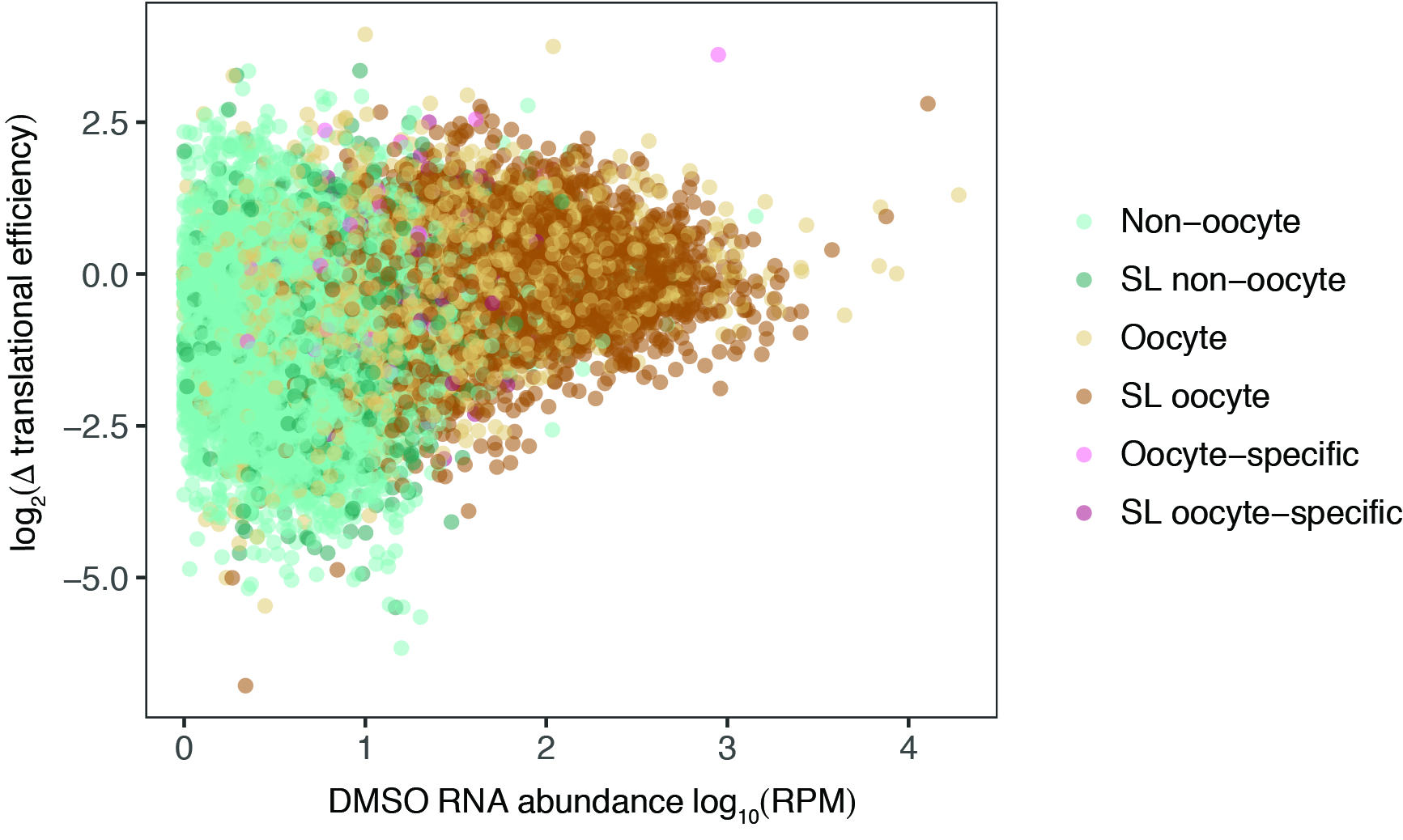

Supplement: Supplementary file 4 — Additional file 4: Figure S4. Oocyte transcripts are trans-spliced and translationally dormant. Changes in translational efficiency in response to Torin 1 (y-axis) against mRNA abundances (RPM = reads per million) in control animals (x-axis) with transcripts categorised as indicated in the legend. [file 12864_2019_6277_MOESM4_ESM.jpg]

a

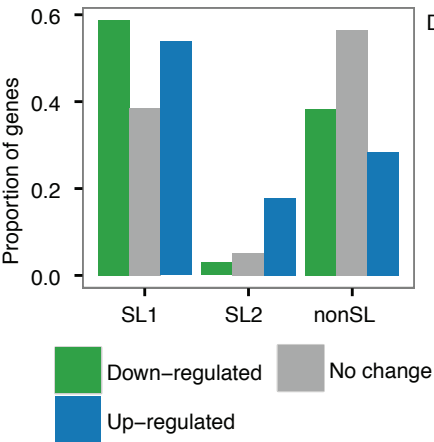

b

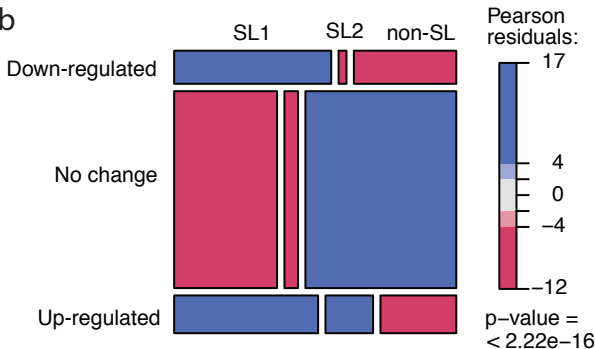

Supplement: Supplementary file 5 — Additional file 5: Figure S5. Translational control during nutrient-dependent recovery from growth arrest is associated with the presence of a 5′ spliced leader in C. elegans. (A) Proportion of genes trans-spliced to SL1 or SL2 or without a spliced leader that have translation up- or down-regulated (or no translational response) upon release from L1 diapause in response to food availability. (B) Mosaic plot shows Pearson residuals from a Chi-square test using genes categorised as in (A). [file 12864_2019_6277_MOESM5_ESM.pdf]

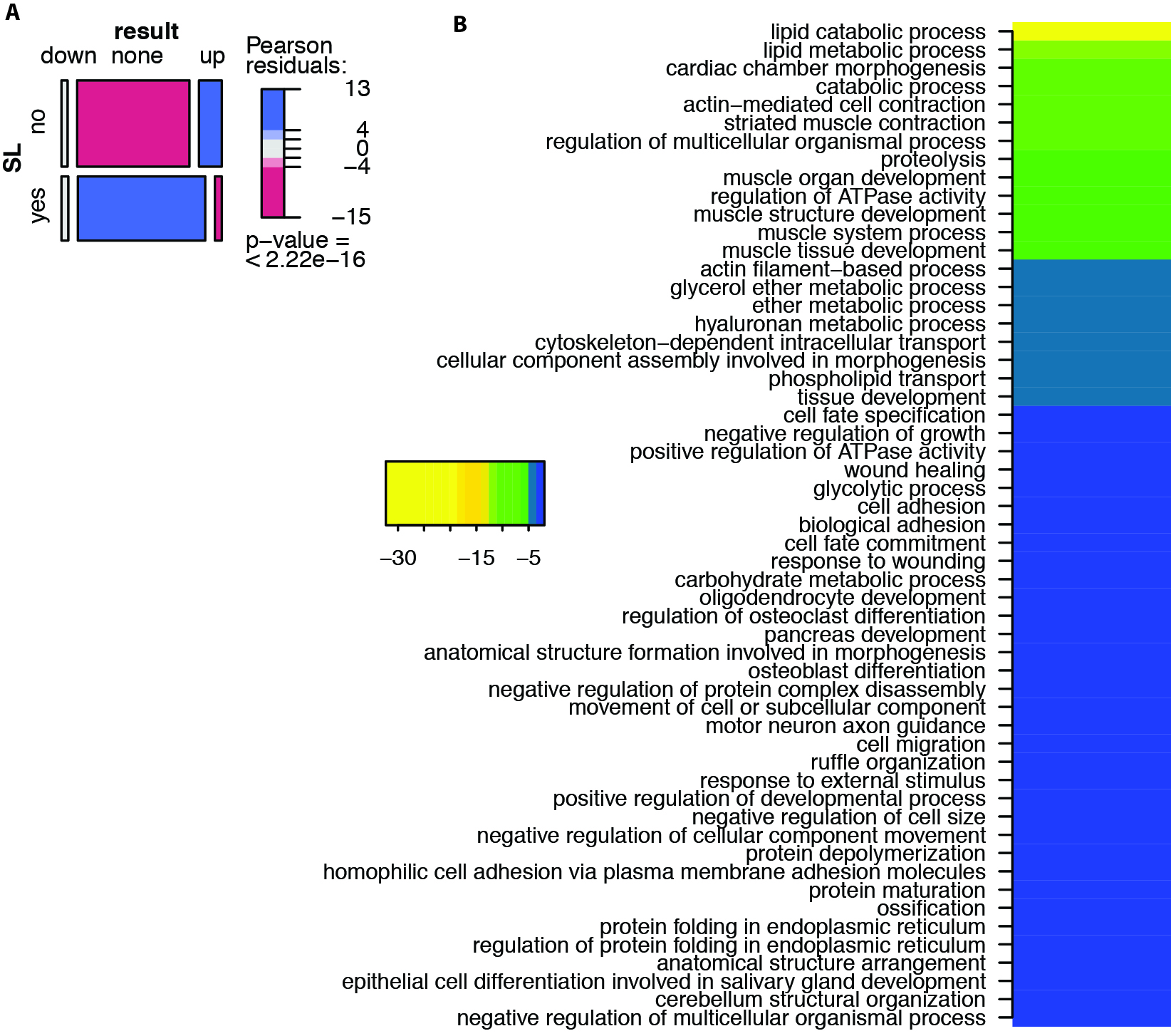

Supplement: Supplementary file 6 — Additional file 6: Figure S6. Transcriptional response during recovery from growth arrest in O. dioica. Genes with significantly up-regulated transcription during recovery from growth arrest were enriched for non-trans-spliced transcripts (A) and GO terms related to lipid metabolism, muscle contraction and proteolysis (B). [file 12864_2019_6277_MOESM6_ESM.jpg]

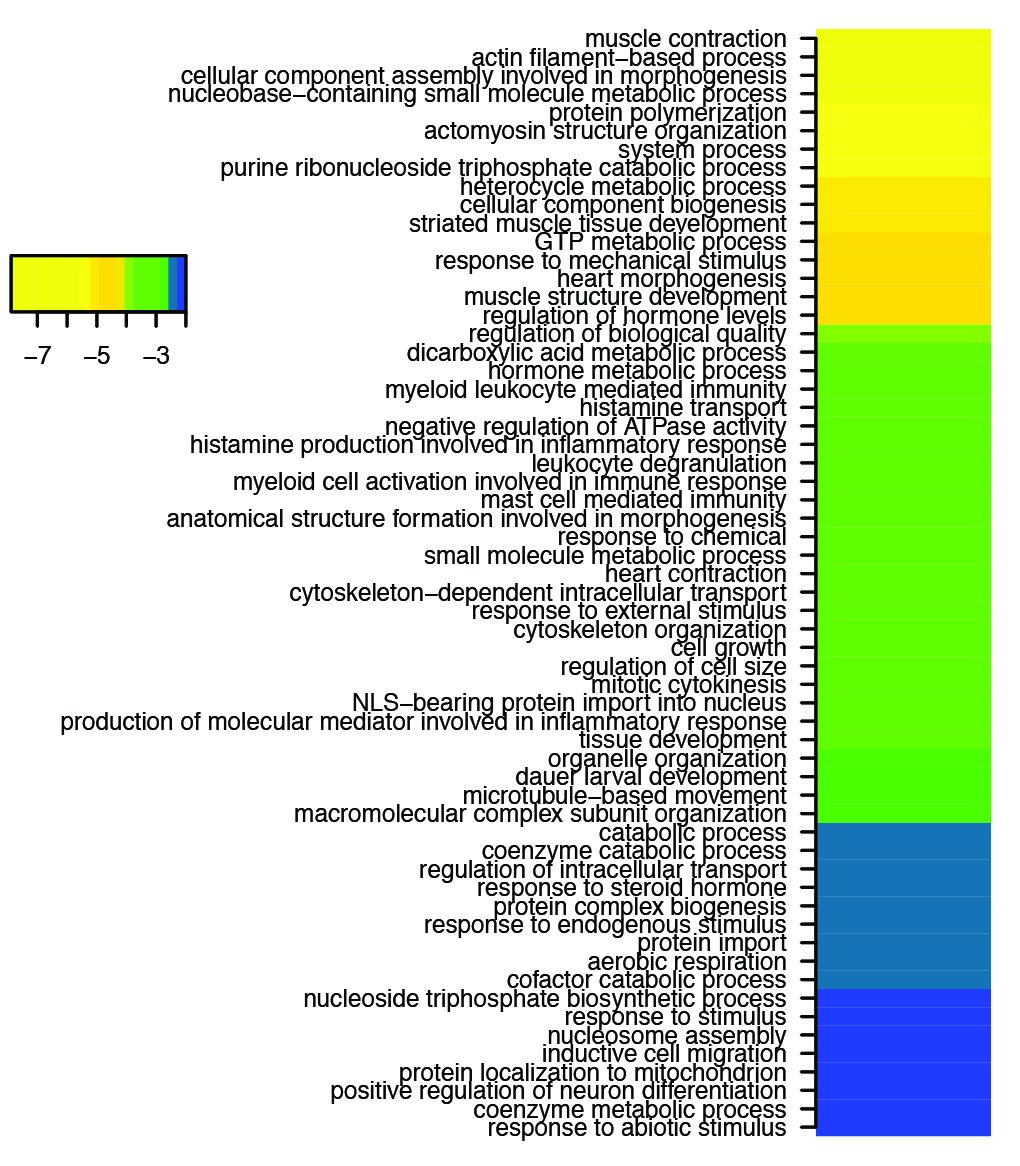

Supplement: Supplementary file 7 — Additional file 7: Figure S7. Translational response during recovery from growth arrest in O. dioica. GO terms enriched in genes with significantly up-regulated translation during recovery from growth arrest. [file 12864_2019_6277_MOESM7_ESM.jpg]
